# Discectomy for Lumbar Disc Herniation in Pediatric and Adolescent Populations: A Systematic Review and Meta-Analysis

**DOI:** 10.7759/cureus.63880

**Published:** 2024-07-05

**Authors:** Christian A Than, Angelique K Valiotis, Abid R Prottoy, Kyle G Alexander, Marios Alogakos, Maamoun Adra, Karen Smayra, Tom J Curtis, Grace E Kim, Hayato Nakanishi, Zaher Dannawi

**Affiliations:** 1 Biomedical Sciences, The University of Queensland, Brisbane, AUS; 2 Pediatrics, University of Nicosia, Limassol, CYP; 3 Neurosurgery, St George's University of London, London, GBR; 4 Neurology, University of Nicosia, Limassol, CYP; 5 General Surgery, St George's University of London, London, GBR; 6 Orthopedics, St George's University of London, London, GBR; 7 Radiology, St George's University of London, London, GBR; 8 Orthopedics, Frimley Health NHS Foundation Trust, Windsor, GBR; 9 Emergency, Swedish Hospital, Chicago, USA; 10 Surgery, St George's University of London, London, GBR; 11 Spine Surgery, Mid and South Essex NHS Foundation Trust, London, GBR

**Keywords:** meta-analysis, odi, vas, herniation, spine, adolescent, pediatric, lumbar, disc, discectomy

## Abstract

Corroborative evidence for discectomy in pediatric or adolescent patients remains scarce, with this single-arm meta-analysis investigating discectomy for lumbar disc herniation (LDH) within this population. PubMed, Embase (Elsevier), CiNAHL, Cochrane Library, Scopus, and Web of Science were searched. Eligible studies reported pediatric patients under 21 years of age with a diagnosis of LDH that was treated surgically with discectomy. This review was registered in PROSPERO (ID: CRD42023463358). Twenty-two studies met the eligibility criteria (n=1182). Visual analog scale (VAS) scores for back pain at baseline were 5.34 (95% CI: 4.48, 6.20, I^2^=98.9%). Postoperative VAS back pain scores after 12 months were 0.88 (95% CI: 0.57, 1.19, I^2^=95.6%). VAS scores for leg pain at baseline were 7.03 (95% CI: 6.63, 7.43, I^2^=93.5%). Postoperative VAS leg pain scores after 12 months were 1.02 (95% CI: 0.68, 1.36, I^2^=97.0%). Oswestry disability index (ODI) scores at baseline were 55.46 (95% CI: 43.69, 67.24, I^2^=99.9%). Postoperative ODI scores after 12 months were 7.82 (95% CI: 4.95, 10.69, I^2^=99.4%). VAS back, VAS leg and ODI scores demonstrated a minimum clinically important difference (MCID) at all postoperative points. Perioperative outcomes demonstrated operative time as 85.71 mins (95% CI: 73.96, 97.46, I^2^=99.4%) and hospital length of stay as 3.81 days (95% CI: 3.20, 4.41, I^2^=98.5%). The postoperative reoperation rate at the same level was 0.01 (95% CI: <0.00, 0.02, I^2^=0%). Discectomy appears safe and effective in pediatric and adolescent patients suffering from LDH. The findings here provide groundwork for future randomized control trials against conservative measures to elaborate on optimal management and elucidate long-term outcomes.

## Introduction and background

Lumbar disc herniation (LDH) is a condition characterized by the displacement of disc material through its external encasing membrane [1]. Its pathological progression is predominantly linked to degenerative disease, involving factors such as compression, tension, shear, and torque stresses, which collectively contribute to this degeneration. These alterations result in the protrusion or herniation through the annulus fibrosus in the central canal, giving rise to clinical symptoms such as nerve root impingement and subsequent sensory motor deficits along the affected pathway [1]. While the most affected demographic comprises of males between the ages of 30 and 50 years, disc herniation, albeit less frequently, can also manifest in the pediatric or adolescent population [2]. Treatment approaches for both pediatric and adult populations often involve conservative management, utilizing physiotherapy and non-steroidal anti-inflammatory drugs (NSAIDs) as primary modalities [1]. However, in cases where symptoms align with refractory conservative measures or compressive spinal emergencies, surgical intervention becomes warranted [1].

In younger populations, cases requiring surgical interventions have been stated as approximately 5.5 per 100,000 persons amongst those under 25 years of age [3]. Current surgical interventions consist of discectomy, in which offending material is surgically removed to alleviate symptoms [4]. Laminectomy is also possible but reserved for more extensive cases [4]. Between the two, discectomy demonstrates more advantageous perioperative outcomes due to its minimally disruptive nature toward the vertebral column [5]. In adult cohorts, discectomy has been shown to result in decreased pain and increased functionality [6]. Pain alleviation following discectomy is achieved through the removal of the offending material that causes nerve compression, in turn minimizing the source of inflammation and immune response [4]. However, due to the lower prevalence, there are limited reports for outcomes of pediatric and adolescent lumbar discectomy in comparison to adult cohorts [7]. This is problematic, as findings in the adult populations may not be directly translated to pediatric or adolescent populations due to growth and a continuously changing skeletal structure [8].

At present, there is a lack of corroborative evidence evaluating the safety and efficacy of discectomy in pediatric populations. Further investigation is needed to evaluate the alleviation of pain and functionality, measured through visual analog scale (VAS) and Oswestry disability index (ODI) scores, respectively. Additionally, elucidation is required regarding perioperative measures such as operative time, estimated blood loss, and hospital length of stay. To date, no other meta-analysis has been conducted on discectomy as a surgical intervention for pediatric and adolescent patients with LDH. As such, this study aims to shed some insight into the overall efficacy of surgical management for pediatric or adolescent populations when treating disc herniation with the current literature state.

## Review

Methods

Search Strategy and Data Sources

This review followed the Preferred Reporting Items for Systematic Reviews and Meta-analyses (PRISMA) guidelines [9]. A comprehensive search was conducted in several databases from their respective inception dates up to September 15, 2023, without any language restrictions. The databases included PubMed, Embase (Elsevier), CiNAHL, Cochrane Central Register of Controlled Trials, Cochrane Database of Systematic Reviews, Scopus, and Web of Science. The search strategy was designed and conducted by a medical reference librarian. Controlled vocabulary supplemented with keywords was used to search for studies describing lumbar discectomy in pediatric or adolescent LDHs. The actual strategy listing all search terms used and how they are combined is available in Supplemental Material 1. Additional references were sought through hand searches of Google Scholar (search terms: Discectomy; Lumbar; Pediatrics; Adolescents; Spine; Herniation). This review was registered prospectively with PROSPERO (CRD42023463358).

Eligibility Criteria and Quality Assessment

Eligible studies must have met all of the following inclusion criteria: 1) patients <21 years of age explicitly classified as either a pediatric and or adolescent population by study authors; 2) discectomy for LDH of the spine (microendoscopic discectomy (MED), percutaneous endoscopic lumbar discectomy (PELD), percutaneous laser disc decompression (PLDD), and tubular discectomy or open discectomy/microdiscectomy); 3) reported on the primary outcomes of VAS back and/or leg pain, ODI, modified MacNab criteria, hospital length of stay, estimated intraoperative blood loss, or operative time; 4) randomized, comparative, case-control, prospective, retrospective, observational cohort or case series in study design. Exclusion criteria were: 1) discectomy was a re-operation of previous surgery; 2) surgery involved laminectomy; 3) case reports, abstracts, conference abstracts, review articles, and letter-to-editors; 4) unpublished data, data published in abstract form only, or non-full-length articles; 5) studies with overlapping patient data. Conventionally, <18 years of age is considered as the pediatric patient. However, the literature demonstrates that growth and ossification of the spine do not end until the second decade of life [10]. Additionally, the acceptance of 21 years of age and under for pediatric inclusion is in accordance with guidelines set by the American Academy of Pediatrics [11]. These two components thus provided the rationale for the age bracket within this meta-analysis.

Article screening and data extraction were conducted by four independent assessors (AKV, ARP, KGA, TJC). Any disagreements were adjudicated by CAT and discussed with co-authors as necessary. The quality of each study was independently evaluated by two authors (ARP and KGA) using the Newcastle Ottawa Assessment Scale [12]. The difference in the determination of quality was resolved by discussion with a third author until a consensus was reached (MA). All included studies were categorized as having one study arm for analysis. Included studies that involved multiple arms had only the arms involving pediatric patients with LDH receiving surgical discectomy extracted. For the purposes of the one-arm meta-analysis, studies that had multiple eligible study arms for extraction (e.g., open discectomy, MED, PELD, PLDD, or microdiscectomy) were all included and treated separately during analysis.

Outcomes

Pain and function were the outcome variables of interest in this meta-analysis. Additional primary outcomes were extracts of operative time in minutes, intraoperative blood loss in milliliters, and hospital length of stay in days. Patient perception of pain was evaluated using the VAS questionnaire score. A lower VAS score indicates less perceived pain, meaning more patient relief [13]. The VAS score was used to evaluate back pain and leg pain separately. VAS scores were for back pain unless specifically stated for leg pain. Patient perception of functionality was evaluated using the ODI scores. A lower ODI score indicates less disability, meaning more patient functionality [13]. Clinician perception of patient symptom relief was evaluated using the modified MacNab criteria [14]. It is the subjective grading of the patient’s perceived quality of surgical outcome using one of four categories: excellent, good, fair, or poor. No perceived pain, along with a return to the original level of activity, would be categorized as an “excellent” outcome. Occasional and localized pain, with relief of presenting symptoms, and return to modified work, would be considered a “good” outcome. Some improvement in functionality and the inability to return to daily activity is a “fair” outcome. Persistent objective symptoms of root involvement, along with the need for additional operative intervention, would be considered a “poor” outcome. Secondary outcomes of anatomical co-morbidities, presenting symptoms, spinal level of herniation, number of levels of operation, herniation classification, prior positive diagnostic tests, preceding trauma, postoperative complication rates (e.g., infection, cerebrospinal fluid (CSF) leak, neurological deficit), lumbar fusion requirement rate and re-operation rate were also extracted.

Statistical Analysis

Means of continuous variables and rates of binary variables were pooled using the generic inverse variance method of DerSimonian and Laird [15]. Proportions underwent logit transformation prior to meta-analysis. The heterogeneity of effect size estimates across the studies was quantified using the Q statistic and the I2 index (P < 0.10 was considered significant) [16]. A value of I2 of 0-25% indicates minimal heterogeneity, 26-50% moderate heterogeneity, and 51-100% substantial heterogeneity. The random-effects model was used. If mean and standard deviation (SD) were unavailable, the median was converted to mean using the formulas from the Cochrane Handbook for Systematic Reviews of Interventions [17]. If SD was not available or extractable, the reported mean was omitted from the calculation. Authors were contacted three times to obtain any relevant additional information that was omitted in published articles. Publication bias was assessed using a funnel plot [18]. Data analysis for a single-arm meta-analysis was performed using Open Meta analyst software (CEBM, Brown University, Providence, USA). Data for the pediatric lumber discectomy group from any multiple-arm studies were included in the one-arm study analyses of the current meta-analysis.

Data Extraction and Minimal Clinical Important Difference (MCID) Interpretation

The VAS and ODI scores were extracted in the following epochs: baseline (preoperation), up to 1 week, 1 month, 3 months, 6 months, and 12 months post-operation. A VAS score of 1.2 and 1.6 was considered as the minimum clinically important difference (MCID) in spine surgery patients with back pain and leg pain, respectively, as previously reported [19]. An ODI score of 8.2 was considered as the MCID as previously reported [19].

Results

**Figure 1:**
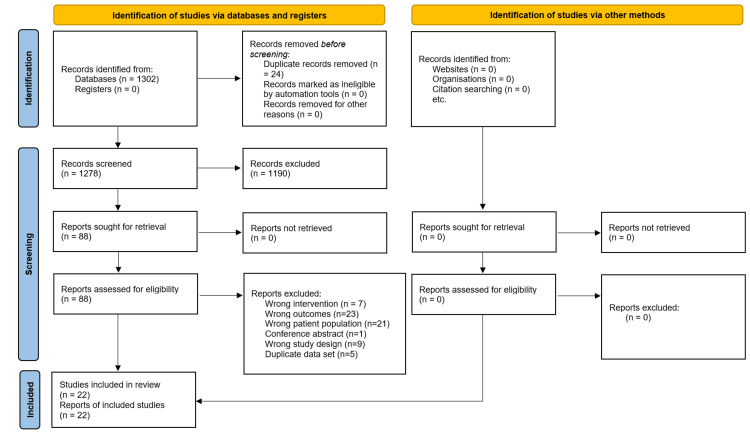
Preferred reporting items for systematic reviews and meta-analyses (PRISMA) flow diagram

The initial search yielded 1302 potentially relevant articles, from which 22 unique studies involving 1182 patients met the eligibility criteria [20-41]. Two studies required translation from Chinese into English for inclusion [26,41]. The PRISMA flow chart illustrates the details of the study selection process in Figure 1. The clinical characteristics of each included study are comprehensively described in Table 1. Of the articles reporting on VAS back score gender, 64.72% (n=554) were male and 35.28% (n=302) were female [20-25,31,33-40]. Of the articles reporting on VAS leg score gender, 63.70% (n=358) were male and 36.30% (n=204) were female [23,25,27,31,33,35-41]. Of the articles reporting on ODI gender, 63.39% (n=419) were male and 36.61% (n=242) were female [21,23,25-27,31,33,35,36,38,39].

**Table 1:** Clinical characteristics of studies a and b denote the same study that was extracted as separate groups for the purpose of meta-analysis, hence the description of 27 studies within this table. Overall, 22 unique separate studies were included in this meta-analysis. FIED: full endoscopic interlaminar discectomy; MED: micro-endoscopic discectomy; NR: not reported; PELD: percutaneous endoscopic lumbar discectomy; PIED: percutaneous interlaminar endoscopic discectomy; PTED: percutaneous transforaminal endoscopic discectomy; SD: standard deviation; OLD: open laminectomy discectomy

Study	Country	Intervention	Patients (n)	Males: Females (n)	Mean age ± SD (years)	Average follow-up ± SD (months)	Study design
Celik et al., 2011 [20]	Turkey	Microdiscectomy	32	14:18	15.00 ± 3.20	83.00 ± 29.75	Prospective cohort study
Erdag et al., 2022 [21]	Turkey	Microdiscectomy	18	11:7	17.10 ± 1.00	36.80 ± 6.00	Retrospective case series
Guclu et al., 2022 [22]	Turkey	Microdiscectomy	9	3:6	14.60 ± 0.94	NR	Retrospective case series
Jie et al., 2015 [26]	China	PELD	30	20:10	17.90 ± 0.75	12.00 ± 1.00	Retrospective comparative study
Lagerback et al., 2015 [23]	Sweden	Unspecified discectomy	151	75:76	17.80 ± 1.50	21.60 ± 3.00	Prospective observational study
Lee et al., 2006 [24]	Korea	PELD	46	26:20	16.50 ± 1.25	37.20 ± 5.75	Retrospective case series
Li et al., 2018a [25]	China	MED	30	20:10	19.40 ± 1.50	67.07 ± 6.76	Retrospective cohort study
Li et al., 2018b [25]	China	PELD	48	30:18	18.96 ± 1.99	68.87 ± 7.03	Retrospective cohort study
Liu et al., 2019 [27]	China	PTED	43	24:19	18.02 ± 1.67	18.33 ± 3.24	Retrospective case series
Mao et al., 2021 [28]	China	PELD	16	11:5	18.70 ± 1.75	17.30 ± 15.00	Retrospective case series
McAvoy et al., 2019 [29]	USA	Microdiscectomy	199	88:111	16.00 ± 1.50	98.40 ± 54.90	Retrospective case series
Montejo et al., 2018 [30]	USA	Microdiscectomy	12	5:7	17.00 ± 1.60	26.40 ± 17.40	Retrospective cohort study
Qu et al., 2023a [31]	China	PIED/PTED	19	12:7	16.50 ± 2.60	12.00 ± 0.00	Prospective cohort study
Qu et al., 2023b [31]	China	PEID/PTED	28	15:13	15.70 ± 2.40	12.00 ± 0.00	Prospective cohort study
Thomas et al., 2011 [32]	USA	Microdiscectomy	6	2:4	16.00 ± 0.75	10.20 ± 5.30	Retrospective case series
Tu et al., 2018a [33]	China	FEID	28	20:8	17.80 ± 3.50	36.50 ± 8.20	Retrospective cohort study
Tu et al., 2018b [33]	China	PIED	46	35:11	18.10 ± 4.20	38.30 ± 7.80	Retrospective cohort study
Wang et al., 2013 [34]	China	MED, PELD, OLD	121	95:26	17.90 ± 1.90	NR	Retrospective case series
Wang et al., 2014 [35]	China	PELD	29	21:8	15.90 ± 1.25	NR	Prospective case series
Wang et al., 2023a [41]	China	PELD	26	15:11	18.56 ± 2.11	3.00 ± 0.00	Retrospective cohort study
Wang et al., 2023b [41]	China	OLD	24	12:12	18.03 ± 2.62	3.00 ± 0.00	Retrospective cohort study
Wu et al., 2023 [36]	China	PEID/PETD	31	24:7	19.19 ± 1.94	12.00 ± 0.00	Retrospective cohort study
Yamaya et al., 2020 [37]	Japan	PELD	18	14:4	17.00 ± 0.75	27.00 ± 6.75	Retrospective case series
Yu et al., 2021a [38]	China	PELD	78	57:21	17.99 ± 2.72	32.31 ± 17.70	Retrospective cohort study
Yu et al., 2021b [38]	China	Open discectomy	40	31:9	18.03 ± 2.19	38.67 ± 16.53	Retrospective cohort study
Zhang et al., 2019 [39]	China	Open discectomy	42	24:18	17.60 ± 1.25	28.20 ± 9.20	Retrospective case series
Zheng et al., 2016 [40]	China	PELD	12	7:5	12.60 ± 1.25	12.00 ± 0.00	Prospective case series

Risk of Bias

Results of the quality assessment of all included studies are shown in Table 2. The studies were judged to be of good quality [20-41]. The exposure and outcome were adequately ascertained, and the lengths of follow-up were adequate to manifest a change in the clinical outcomes.

**Table 2:** Study quality assessment using the Newcastle Ottawa scale Good quality: 3 or 4 stars (*) in the selection domain AND 1 or 2 stars in the comparability domain AND 2 or 3 stars in the outcome domain; fair quality: 2 stars in the selection domain AND 1 or 2 stars in the comparability domain AND 2 or 3 stars in the outcome/exposure domain; poor quality: 0 or 1 star in selection domain OR 0 stars in comparability domain OR 0 or 1 stars in outcome/exposure domain N/A: No association

Study	Selection				Comparability	Outcome			Quality score
	Representativeness of the exposed cohort	Selection of the non-exposed cohort	Ascertainment of exposure	Demonstration that outcome of interest was not present at start of study	Comparability of cohorts on the basis of design or analysis	Assessment of outcome	Was follow-up long enough for outcomes to occur	Adequacy of follow-up of cohorts	
Celik et al., 2011 [20]	A*	N/A	A*	A*	N/A	B*	A*	A*	6
Erdag et al., 2022 [21]	A*	N/A	A*	A*	N/A	B*	A*	A*	6
Guclu et al., 2022 [22]	A*	N/A	A*	A*	N/A	B*	A*	A*	6
Jie et al., 2015 [26]	A*	N/A	A*	A*	N/A	B*	A*	A*	6
Lagerback et al., 2015 [23]	A*	N/A	A*	A*	N/A	B*	A*	A*	6
Lee et al., 2006 [24]	B*	N/A	A*	A*	N/A	B*	A*	D	5
Li et al., 2018 [25]	A*	N/A	A*	A*	N/A	B*	A*	A*	6
Liu et al., 2019 [27]	A*	N/A	A*	A*	N/A	B*	A*	A*	6
Mao et al., 2021 [28]	A*	N/A	A*	A*	N/A	B*	A*	A*	6
McAvoy et al., 2019 [29]	A*	N/A	A*	A*	N/A	B*	A*	A*	6
Montejo et al., 2018 [30]	A*	N/A	A*	A*	N/A	B*	A*	C	5
Qu et al., 2023 [31]	B*	N/A	A*	A*	N/A	B*	A*	A*	6
Thomas et al., 2011 [32]	A*	N/A	A*	A*	N/A	B*	A*	A*	6
Tu et al., 2018 [33]	B*	N/A	A*	A*	N/A	B*	A*	A*	6
Wang et al., 2013 [34]	A*	N/A	A*	A*	N/A	B*	A*	A*	6
Wang et al., 2014 [35]	A*	N/A	A*	A*	N/A	B*	A*	A*	6
Wang et al., 2023 [41]	A*	N/A	A*	A*	N/A	B*	A*	A*	6
Wu et al., 2023 [36]	A*	N/A	A*	A*	N/A	B*	A*	A*	6
Yamaya et al., 2020 [37]	B*	N/A	A*	A*	N/A	B*	A*	A*	6
Yu et al., 2021 [38]	A*	N/A	A*	A*	N/A	B*	A*	A*	6
Zhang et al., 2019 [39]	B*	N/A	A*	A*	N/A	B*	A*	A*	6
Zheng et al., 2016 [40]	A*	N/A	A*	A*	N/A	B*	A*	A*	6

Preoperative Outcomes

Preoperative outcomes have been reported in Table 3. There were no reports of operative levels of L1/L2 [20-37,40,41], 2 reports of operation on L2/L3 [20-37,40,41], 67 reports of operation on L3/L4 [20-37,40,41], 647 reports of operation on L4/L5 [20-41] and 498 reports of operation on L5/S1 [20-41]. Out of 1182 patients reported for operative levels, 1129 were 1-level operations [20,21,24-41] and 53 were 2-level operations [20,21,24-41].

**Table 3:** Preoperative outcomes CT: computed tomography; MRI: magnetic resonance imaging

	Proportion	95% CI	Events	I^2^	Included study groups (N)	Sample size (N)
Operative level						
L1/L2	0.01	0.00-0.01	0	0%	24	1022
L2/L3	0.01	0.00-0.01	2	0%	24	1022
L3/L4	0.04	0.02-0.06	67	72.8%	24	1022
L4/L5	0.53	0.47-0.60	647	78.4%	27	1182
L5/S1	0.43	0.35-0.50	498	87.3%	27	1182
Number of operative levels						
1 level	0.94	0.91-0.98	1129	95.6%	27	1182
2 levels	0.06	0.03-0.09	53	95.6%	27	1182
Type of disc herniation						
Bulging	0.03	0.00-0.05	10	24.6%	6	274
Extrusion	0.27	0.17-0.37	62	70.8%	6	274
Protrusion	0.48	0.05-0.91	173	99.3%	6	274
Sequestration	0.23	0.06-0.39	43	94.3%	6	274
Levels of degeneration						
1 level	0.93	0.89-0.97	943	96.0%	25	1022
2 levels	0.07	0.03-0.11	73	96.0%	25	1022
3 levels	0.01	0.01-0.02	5	0%	25	1022
Positive straight leg raise	0.79	0.69-0.88	441	92.9%	11	522
Positive imaging						
CT	0.73	0.40-1.06	132	99.9%	8	448
MRI	0.99	0.99-1.00	799	0%	19	799
Radiography	0.86	0.60-1.12	293	99.8%	8	483
Preceding trauma	0.28	0.17-0.38	122	89.5%	9	576

Out of 274 patients reported for herniation classification, there were 10 reports of bulging [20,33-35,37], 62 reports of extrusion [20,33-35,37], 173 reports of protrusion [20,33-35,37], and 43 reports of sequestration [20,33-35,37]. Furthermore, out of 1022 patients reported for levels of degeneration, there were 943 reports of 1-level degeneration [20,21,24-41], 73 reports of 2-level degeneration [20,33-35,37,41], and 5 reports of 3-level degeneration [20,33-35,37,41].

Out of 522 patients who were tested for straight leg tests, there were reports of 441 positive tests [21,22,27,29,30,32,34-36,39-41]. Moreover, there were 132 reports of positive CT scans [29,32,34-36,40,41], 799 reports of positive MRI scans [20-22,26,27,29-38,40], and 293 reports of positive radiography [29,32-35,39,40]. There were 122 reports of preceding trauma [22,24,29,30,34,35,38,39].

Presenting Symptoms

Presenting symptoms have been reported in Table 4. Of 537 patients, leg pain was reported in 508 patients, back pain was reported in 429 patients, motor weakness/hemiparesis was reported in 158 patients, sensory loss was reported in 148 patients, dysesthesia was reported in 114 patients, tendon reflex loss was reported in 83 patients, reactive scoliosis was reported in 54 patients, neurogenic claudication was reported in 45 patients, bowel/bladder changes were reported in 9 patients, and no hemiplegia was reported in any patients [21,22,24,27,29,30,32,34,35,39,40].

**Table 4:** Presenting symptoms

Presenting symptoms	Proportion	95% CI	Events	I^2^	Included study groups (N)	Sample size (N)
Back pain	0.77	0.64-0.90	429	97.3%	11	537
Bowel/bladder changes	0.01	0.00-0.02	9	0%	11	537
Dysesthesia	0.17	0.00-0.38	114	99.7%	11	537
Hemiplegia	0.00	0.00-0.01	0	0%	11	537
Leg pain	0.96	0.93-0.99	508	59.3%	11	537
Motor weakness/hemiparesis	0.35	0.20-0.50	158	98.0%	11	537
Neurogenic claudication	0.05	0.01-0.08	45	86.3%	11	537
Reactive scoliosis	0.05	0.01-0.09	54	80.1%	11	537
Sensory loss	0.27	0.00-0.55	148	99.7%	11	537
Tendon reflex loss	0.09	0.04-0.15	83	90.4%	11	537

Anatomical Co-morbidities

The anatomical co-morbidities of the patients are presented in Table 5. Out of 726 patients, there were reports of 89 with scoliosis, 32 with stenosis (spinal canal or unspecified), 11 with stenosis (lateral recess), 9 with stenosis (foraminal), 7 with dehydrated discs, 4 with disc calcifications, 4 with disc cysts, 3 with bone hyperplasia, 2 with spondylolisthesis grade I, 2 with lumber instability, 2 with lumbarization, 1 with Bertolotti’s syndrome, 1 with lumbosacral transitional vertebral, 1 with occult spina bifida, 1 with sacralization, 1 with Schmorl’s node, 1 with a Tarlov cyst and no reports of hypertrophy of yellow ligament [20-22,24-30,32-34,39].

**Table 5:** Anatomical co-morbidities

Anatomical co-morbidities	Proportion	95% CI	Events	I^2^	Included study groups (N)	Sample size (N)
Bertolotti’s syndrome	<0.01	<0.01-0.01	1	0%	14	726
Bone hyperplasia	<0.01	<0.01-0.01	3	0%	14	726
Dehydrated disc	<0.01	<0.01-0.01	7	0%	14	726
Disc calcification	0.01	<0.01-0.01	4	0%	14	726
Disc cyst	0.01	<0.01-0.01	4	0%	14	726
Hypertrophy of yellow ligament	<0.01	<0.01-0.01	0	0%	14	726
Occult spina bifida	<0.01	<0.01-0.01	1	0%	14	726
Lumbarization	<0.01	<0.01-0.01	2	0%	14	726
Lumbar instability	<0.01	<0.01-0.01	2	0%	14	726
Lumbosacral transitional vertebra (LSTV)	<0.01	<0.01-0.01	1	0%	14	726
Sacralization	<0.01	<0.01-0.01	1	0%	14	726
Schmorl’s node	<0.01	<0.01-0.01	1	0%	14	726
Scoliosis	0.21	0.08-0.33	89	99.7%	14	726
Spondylolisthesis grade I	<0.01	<0.01-0.01	2	0%	14	726
Stenosis (foraminal)	<0.01	<0.01-0.01	9	0%	14	726
Stenosis (lateral recess)	<0.01	<0.01-0.01	11	0%	14	726
Stenosis (spinal canal or unspecified)	0.02	<0.01-0.04	32	66.3%	14	726
Tarlov cyst	<0.01	<0.01-0.01	1	0%	14	726

Visual Analog Scale (VAS)

VAS scores for back and leg pain, from baseline to follow-up, are listed in Table 6. The average baseline pain scores of VAS back pain were 5.34. Up to one week, postoperative pain scores were 2.55. At one month postoperative pain scores were 1.87. At three months postoperative pain scores were 1.31. At six months postoperative pain scores were 1.20. At 12 months postoperative pain scores were 0.88. The values are depicted in Supplemental Material 2 and their associated funnel plots are in Supplemental Material 3 [20-25,31,33-40]. Although visual inspection of funnel plots indicated asymmetry for time points 3 months, 6 months, and 12 months, the inclusion of less than 10 studies at each point limited distinguishing chance from real asymmetry. MCID was achieved at all time points and is listed in Table 7.

**Table 6:** Primary outcomes ODI: Oswestry disability index; Post-op: postoperative; VAS: visual analog score

Outcomes					
	Mean	95% CI	I^2^	Included study groups (N)	Sample size (N)
VAS score (back/unspecified pain)					
Baseline	5.34	4.48-6.20	98.9%	20	733
Post-op up to first week	2.55	1.96-3.14	98.1%	11	385
Post-op 1 month	1.87	1.38-2.36	92.9%	5	126
Post-op 3 months	1.31	0.76-1.86	98.0%	10	348
Post-op 6 months	1.20	0.92-1.47	92.7%	8	259
Post-op 12 months	0.88	0.57-1.19	95.6%	7	211
VAS score (leg pain)					
Baseline	7.03	6.63-7.43	93.5%	15	589
Post-op up to first week	1.86	1.40-2.33	95.0%	7	228
Post-op 1 month	1.64	1.36-1.93	67.0%	3	76
Post-op 3 months	1.34	1.00-1.69	87.1%	5	150
Post-op 6 months	1.02	0.72-1.31	95.8%	9	302
Post-op 12 months	1.02	0.68-1.36	97.0%	8	236
ODI					
Baseline	55.46	43.69-67.24	99.9%	15	661
Post-op up to first week	14.02	10.35-17.69	98.3%	4	152
Post-op 1 month	20.94	15.39-26.50	95.1%	3	76
Post-op 3 months	14.19	10.65-17.73	98.7%	8	298
Post-op 6 months	10.24	8.39-12.10	97.2%	9	302
Post-op 12 months	7.82	4.95-10.69	99.4%	8	254

**Table 7:** Minimal clinically important difference A VAS score change of 1.2 and 1.6 was considered as the MCID in spinal surgery with back pain and leg pain, respectively. An ODI score change of 8.2 was considered as the MCID. *: reaches MCID BL: baseline; MCID: minimal clinically important difference; ODI: Oswestry disability index; Post-op: postoperative; VAS: visual analog score

MCID	BL - 1 week post-op	BL - 1 month post-op	BL - 3 months post-op	BL - 6 months post-op	BL - 12 months post-op
VAS score (back/unspecified pain) mean difference	5.17*	5.39*	5.69*	6.01*	6.01*
VAS score (leg pain) mean difference	2.79*	3.47*	4.03*	4.14*	4.46*
ODI mean difference	41.44*	34.52*	41.27*	45.22*	47.64*

The average baseline pain scores of VAS leg pain were 7.03. Up to one week, postoperative pain scores were 1.86. At one month postoperative pain scores were 1.64. At three months postoperative pain scores were 1.34. At six months postoperative pain scores were 1.02. At 12 months postoperative pain scores were 1.02. The values are depicted in Supplemental Material 4 and their associated funnel plots in Supplemental Material 5 [23,25,27,31,33,35-41]. Although visual inspection of funnel plots indicated asymmetry for all epochs, the inclusion of fewer than 10 studies at each point limited distinguishing chance from real asymmetry. MCID was achieved at all time points and is listed in Table 7.

Oswestry Disability Index (ODI)

Baseline ODI and follow-up are listed in Table 6. The average baseline ODI was 55.46. Up to one week postoperative ODI was 14.02. At one month postoperative ODI was 20.94. At three months postoperative ODI was 14.12. At six months postoperative ODI was 10.24. At 12 months postoperative ODI was 7.82. The values are depicted in Supplemental Material 6 and their associated funnel plots are in Supplemental Material 7 [21,23,25-27,31,33,35,36,38,39]. Although visual inspection of funnel plots indicated asymmetry for time points 3 months, 6 months, and 12 months, the inclusion of fewer than 10 studies at each point limited distinguishing chance from real asymmetry. MCID was achieved at all time points and is listed in Table 7.

Perioperative Outcomes

The perioperative outcomes are listed in Table 8. Operative time (minutes) was 85.71. Estimated intraoperative blood loss (ml) was 139.99. Hospital length of stay (days) was 3.81. The values are depicted in Supplemental Material 8 and their associated funnel plots in Supplemental Material 9 [22,25,26,29-36,38,41]. Visual inspection of funnel plots indicated potential asymmetry for operative time and estimated intraoperative blood loss.

**Table 8:** Perioperative outcomes Min: minutes

Perioperative outcomes	Mean	95% CI	I^2^	Included study groups (N)	Sample size (N)
Operative time (min)	85.71	73.96-97.46	99.4%	20	664
Estimated intraoperative blood loss (ml)	139.99	107.13-172.85	100%	14	645
Hospital length of stay (days)	3.81	3.20-4.41	98.5%	20	804

Postoperative Outcomes and MacNab Scores

The postoperative and McNab clinical outcome scores are listed in Table 9. Lumber fusion was required for two patients [24-26,29,38]. Reoperation of the same level was conducted in 11 patients [24-26,29-31,34-36,38-40]. The values are depicted in Supplemental Material 10 and their associated funnel plots are in Supplemental Material 11. Visual inspection of funnel plots indicated potential asymmetry for both outcomes; however, the inclusion of fewer than 10 studies for lumbar fusion limited distinguishing chance from real asymmetry.

**Table 9:** Postoperative outcomes and MacNab criteria

Outcomes	Proportion	95% CI	Events	I^2^	Included study groups (N)	Sample size (N)
Postoperative						
Lumbar fusion requirement	<0.01	2	0%	7	471
Reoperation same level	0.01	<0.00-0.02	11	0%	15	765
MacNab criteria						
Excellent	0.53	0.42-0.64	293	87.2%	15	561
Good	0.32	0.23-0.42	205	84.4%	15	561
Fair	0.03	0.01-0.04	23	19.8%	15	561
Poor	0.01	<0.00-0.03	7	0%	15	561

Of 561 total MacNab patient reports, 293 were reported as excellent, 205 were reported as good, 23 were reported as fair and 7 were reported as poor. The values are depicted in Supplemental Material 12 and their associated funnel plots in Supplemental Material 13 [22,24,27,28,30,33-36,38,40]. Visual inspection of funnel plots indicated potential asymmetry for scores of fair and poor.

Complications

Complications of the operations are reported in Table 10. There were reports of 15 residual disc herniation/recurrence [24-41], 10 dysesthesia cases [22,24-41], 5 infections [22,24-41], 5 postoperative CSF leaks [24-41], 5 progressive disc degenerations [24-41], 4 postoperative leg pain reports [24-27,29-41], 3 postoperative numbness reports [24-41], 3 intraoperative CSF leaks [22,24-41], 2 nerve root damage reports [24-27,29-40], 2 postoperative neurological deficit cases [24-41], 2 vessel injury reports [24-27,29-41], 1 dural tear case [22,24-41], 1 poor wound healing case [24-41] and no reports of bowel/bladder symptoms [22,24-41].

**Table 10:** Complications CSF: cerebrospinal fluid; Post-op: postoperative

Complications	Proportion	95% CI	Events	I^2^	Included study groups (N)	Sample size (N)
Bowel/bladder symptoms	<0.01	<0.01-0.01	0	0%	24	981
Dural tear	<0.01	<0.01-0.01	1	0%	24	981
Dysesthesia	<0.01	<0.01-0.01	10	0%	24	981
Infection	0.01	<0.01-0.02	5	0%	24	981
Intraoperative CSF leak	0.01	<0.01-0.02	3	0%	23	972
Nerve root damage	<0.01	<0.01-0.01	2	0%	22	956
Post-op CSF leak	0.01	<0.01-0.02	5	0%	23	972
Post-op leg pain	<0.01	<0.01-0.01	4	0%	22	956
Post-op neurological deficit	<0.01	<0.01-0.01	2	0%	23	972
Post-op numbness	<0.01	<0.01-0.01	3	0%	23	972
Poor wound healing	<0.01	<0.01-0.01	1	0%	23	972
Progressive disc degeneration	<0.01	<0.01-0.01	5	0%	23	972
Residual disc herniation/recurrence	0.02	<0.01-0.03	15	0%	23	972
Vessel injury	<0.01	<0.01-0.01	2	0%	22	956

Discussion

The role of discectomy in the management of pediatric and adolescent LDH has steadily garnered emerging literature. The current study is the first meta-analysis to corroborate existing evidence to determine the safety and efficacy of discectomy in treating children with LDH. Our findings suggest that discectomy will improve pain and functionality promptly after surgery, with near resolution up to 12 months postoperatively. At all postoperative follow-up points, an MCID is achieved. Perioperative outcomes demonstrate a relatively short surgical time and corresponding hospital stay. Postoperative complications appear minimal, with low rates of reoperation at the same level. These findings, although preliminary, suggest that discectomy appears to be viable in certain pediatric and adolescent patients suffering LDH who are refractory to conservative measures.

Employment of conservative measures has demonstrated a 63% resorption rate by Wang et al. [42] in adult populations. However, pediatric LDH is less likely to resorb [43]. Limited high-level evidence exists on the incidence of resorption of pediatric LDH, with a scarcity of studies reporting this outcome. Hence, indications for pediatric and adolescent surgery, through refractory conservative measures, warrant valid consideration based on unlikely recovery without intervention. Pediatric surgery is indicated at 4-6 weeks if severe pain persists [42]. Conflicting data exists on the comparison of the long-term outcomes of conservative versus surgical treatment. However, it is broadly accepted that nonsurgical management is not as effective in children as in adults, with a recent review reporting that the short- to long-term effectiveness of conservative treatment for pediatric LDH without neurological deficits varied from 25% to 50% [14]. A randomized control trial by Bailey et al. [44] concluded that surgical treatment was superior to conservative management for sciatica secondary to LDH, lasting for 4-12 months. A significant improvement in leg pain intensity score was reported at six months. The long-term benefits of surgical intervention remain scarce, although existing studies report similar pain and disability outcomes [4]. In particular, a prospective cohort study evaluating the 10-year outcomes reported a more complete relief of leg pain and improved function and satisfaction with surgical management [45]. Overall, it appears that discectomy is more effective in treating pediatric LDH [43]. As such, some studies advocate for earlier surgical intervention in pediatric and adolescent LDH when compared to treating adults. It is suggested that such proposed approaches would lead to a decreased period of disability and streamline return to school and normal activities [46].

Both the patient's family and surgeon's apprehension to surgically manage LDH can arise from the perceived benefits and risks of the procedure. Although rare, both sides should be aware of the potential complications of discectomy. Such complications include but are not limited to, infection and postoperative neurological deficits with associated nerve root damage. However, the results of the current meta-analysis demonstrate considerably low complication rates, which are substantiated by a recent literature review also reporting low complication rates (1.0%-2.6%) [47]. This is further complemented by low rates of recurrence and reoperation seen within the current meta-analysis results. The findings presented here, along with the achievement of MCID for pain and functionality, demonstrate support for discectomy as a safe and effective option for the management of pediatric LDH where needed.

Although the mechanism of pain relief through discectomy remains the same as in adults, careful consideration must account for the differences in pediatric and adolescent spinal anatomy. In particular, the vertebral canal and its associated discs may be smaller thus making the operation more technically challenging. Recent literature lacks studies that have examined the occurrence of disc disease in the pediatric population while considering the influence of skeletal maturity and hormonal changes on the developing spine [48]. As children grow older, substantial changes occur in the composition and shape of their joints, as well as in the consistency of the intervertebral disc. These changes play a crucial role in the response of the spine to injury and in the effectiveness of both conservative and surgical treatments [48].

Spinal surgery at a young age sparks significant concerns regarding the future prognosis of these patients over the span of several decades. However, at present, the long-term effects of discectomy into adulthood for pediatric LDH surgeries have not been extensively studied, and as such, further investigation is required. A retrospective cohort study analyzing the long-term outcomes of discectomy in pediatric LDH, before the advent of microdiscectomy and minimally invasive discectomy, revealed that at a 20-year follow-up from the initial operation, approximately 25% of patients underwent another discectomy, with an additional 5% undergoing arthrodesis [49]. A similar study, reporting the long-term outcomes of discectomy for pediatric LDH, concluded that surgical management does not appear to lead to chronic back pain, or negatively impact overall health, based on a mean follow-up of 8.5 years [50]. At present, there is lacking comparable data from studies evaluating more modern surgical techniques. It is crucial to note that in the long-term, it remains unknown whether adolescents are at an increased risk of future spinal surgery by choosing surgical intervention over conservative management [47]. Limited by the availability of reported long-term outcomes, our study was only able to report outcomes up to 12 months post-discectomy.

Limitations

As with all meta-analyses, limitations are present within the current study. Foremost is the high heterogeneity in outcomes, suggesting a cautionary approach to the interpretation of results. The heterogeneity can likely be attributed to the relatively small sample size and diversity in both surgical methodology and the pediatric and adolescent population across the included studies, as well as the difference in follow-up periods. This potentially limits the generalization of these results to a broader population and prohibits investigation into long-term outcomes. Thus, long-term multicenter studies, with wide-encompassing patient demographics, healthcare settings, and clinical practices would offer a more comprehensive understanding of pediatric discectomy. Second, the lack of randomized control trials within the literature prevented any form of two-arm analysis from further validating the results of the current study. Coupled with the predominantly retrospective design of the included studies, inherent challenges exist within the current meta-analysis in mitigating patient-selection bias. However, the current state of literature predominates with retrospective case series, cohort studies, and prospective observational studies rather than randomized controlled trials; hence, the nature of the overall quality of evidence found within this meta-analysis.

Third, a lack of reporting on failed preoperative conservative measures prohibited any insight into the management employed prior to the requirement for surgery. This may include epidural steroids or selective nerve root injections which would be considered non-operative modalities. Fourth, a lack of reporting regarding postoperative rehabilitative measures precluded further discussion on the influence this may have had on outcomes. Fifth, for ethical reasons, patients could not be randomized to further supplemental treatments such as analgesics or undocumented therapies, and a lack of adequate reporting prevented controlling for their effects. Sixth, not all studies controlled for surgeon experience, which may have influenced outcomes. Finally, the MCID used within the current study was based on literature calculated in adults due to lacking reports on children. Thus the cut-offs may not have the same application in pediatric populations and should be acknowledged.

## Conclusions

The current work is the first meta-analysis to examine the safety and efficacy of discectomy for pediatric and adolescent patients with LDH. These findings suggest discectomy may improve patient perception of pain and functionality as measured by VAS and ODI scores, as well as the McNab criteria. The resolution of symptoms appears to manifest within the first week after surgery, with an almost sequential remission up to 12 months postoperatively. Both pain and functionality are seen to achieve MCID at all time points postoperatively. Postoperative complications appear minimal with a low reoperation rate. Therefore, in pediatric and adolescent patients who are refractory to conservative treatment for LDH, discectomy appears plausible as a therapeutic intervention. Further randomized studies against conservative management are required with higher sample sizes, standardized discectomy protocols, and longer follow-up times to elucidate the findings of this study.

## Figures and Tables

**Figure 2:**
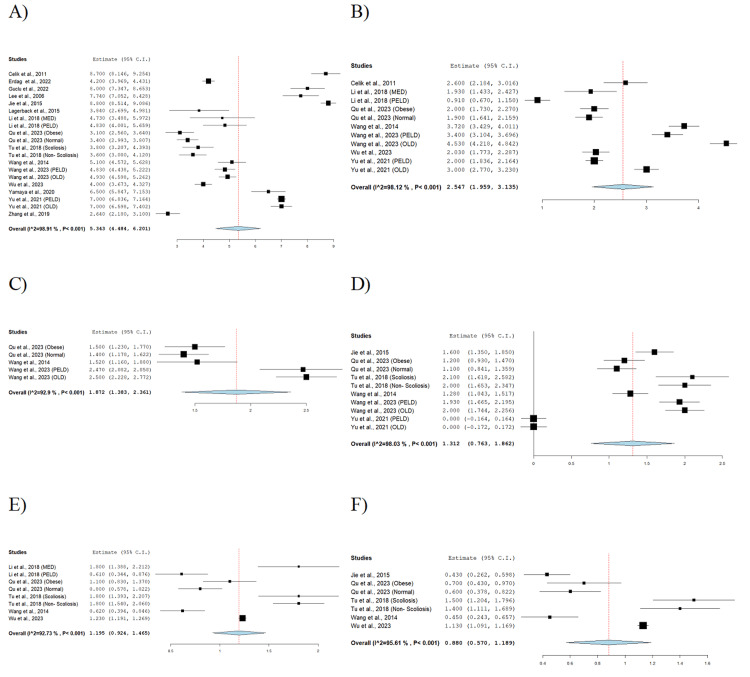
Visual analog scale (VAS) back or unspecified pain A) baseline; B) up to 1 week post-operation; C) 1 month post-operation; D) 3 months post-operation; E) 6 months post-operation; F) 12 months post-operation Reference: Celik et al., 2011 [20]; Erdag et al., 2022 [21]; Guclu et al., 2022 [22]; Lee et al., 2006 [24]; Jie et al., 2015 [26]; Lagerback et al., 2015 [23]; Li et al., 2018 (MED) [25]; Li et al., 2018 (PELD) [25]; Qu et al., 2023 (Obese) [31]; Qu et al., 2023 (Normal) [31]; Tu et al., 2018 (Scoliosis) [33]; Tu et al., 2018 (Non-Scoliosis) [33]; Wang et al., 2014 [35]; Wang et al., 2023 (PELD) [41]; Wang et al., 2023 (OLD) [41]; Wu et al., 2023 [36]; Yamaya et al., 2020 [37]; Yu et al., 2021 (PELD) [38]; Yu et al., 2021 (OLD) [38]; Zhang et al., 2019 [39] PELD: percutaneous endoscopic lumbar discectomy; MED: microendoscopic discectomy; OLD: open laminectomy discectomy

**Figure 3:**
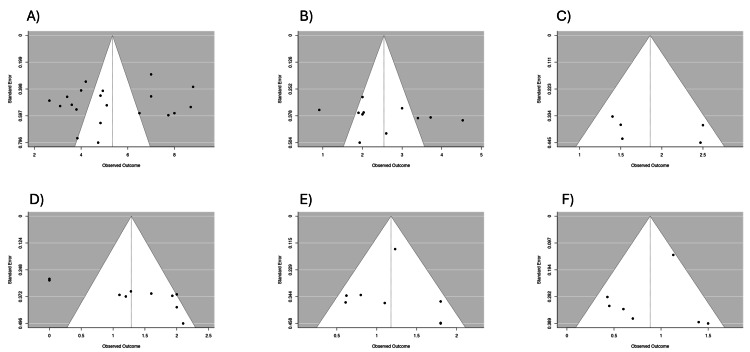
Funnel plots for visual analog scale (VAS) back or unspecified pain A) baseline; B) up to 1 week post-operation; C) 1 month post-operation; D) 3 months post-operation; E) 6 months post-operation; F) 12 months post-operation

**Figure 4:**
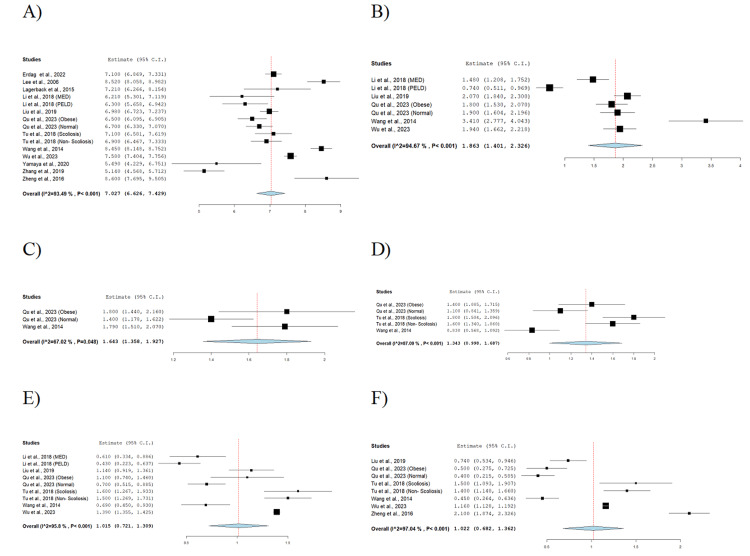
Visual analog scale (VAS) leg pain A) baseline; B) up to 1 week post-operation; C) 1 month post-operation; D) 3 months post-operation; E) 6 months post-operation; F) 12 months post-operation Reference: Erdag et al., 2022 [21]; Lee et al., 2006 [24]; Lagerback et al., 2015 [23]; Li et al., 2018 (MED) [25]; Li et al., 2018 (PELD) [25]; Liu et al., 2019 [27]; Qu et al., 2023 (Obese) [31]; Qu et al., 2023 (Normal) [31]; Tu et al., 2018 (Scoliosis) [33]; Tu et al., 2018 (Non-Scoliosis) [33]; Wang et al., 2014 [35]; Wu et al., 2023 [36]; Yamaya et al., 2020 [37]; Zhang et al., 2019 [39]; Zheng et al., 2016 [40] PELD: percutaneous endoscopic lumbar discectomy; MED: microendoscopic discectomy; OLD: open laminectomy discectomy

**Figure 5:**
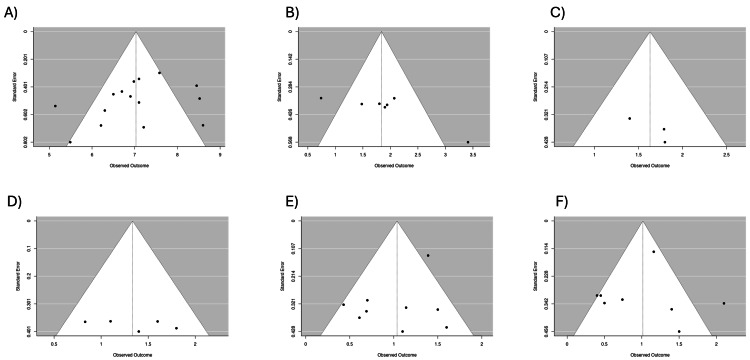
Funnel plots visual analog scale (VAS) leg pain A) baseline; B) up to 1 week post-operation; C) 1 month post-operation; D) 3 months post-operation; E) 6 months post-operation; F) 12 months post-operation

**Figure 6:**
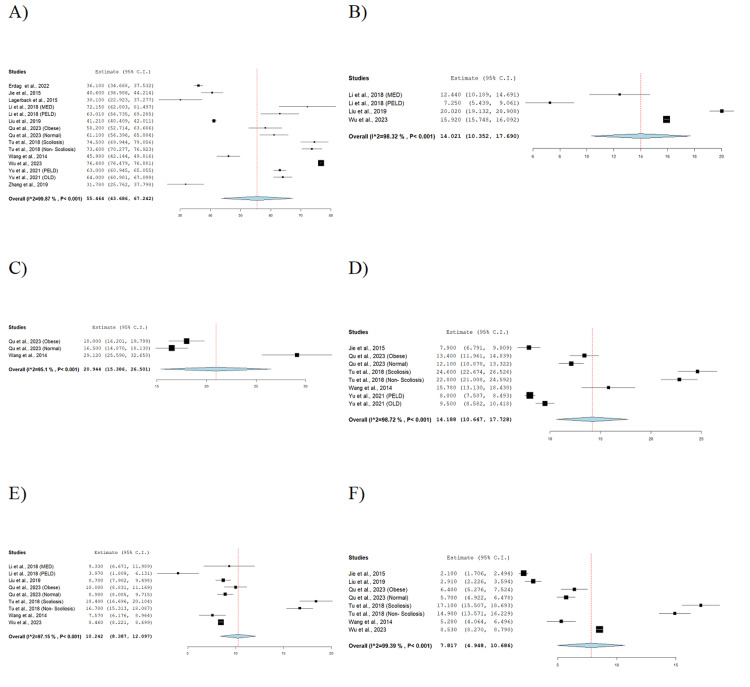
Oswestry disability index (ODI) A) baseline; B) up to 1 week post-operation; C) 1 month post-operation; D) 3 months post-operation; E) 6 months post-operation; F) 12 months post-operation Reference: Erdag et al., 2022 [21]; Jie et al., 2015 [26]; Lagerback et al., 2015 [23]; Li et al., 2018 (MED) [25]; Li et al., 2018 (PELD) [25]; Liu et al., 2019 [27]; Qu et al., 2023 (Obese) [31]; Qu et al., 2023 (Normal) [31]; Tu et al., 2018 (Scoliosis) [33]; Tu et al., 2018 (Non-Scoliosis) [33]; Wang et al., 2014 [35]; Wu et al., 2023 [36]; Yu et al., 2021 (PELD) [38]; Yu et al., 2021 (OLD) [38]; Zhang et al., 2019 [39] PELD: percutaneous endoscopic lumbar discectomy; MED: microendoscopic discectomy; OLD: open laminectomy discectomy

**Figure 7:**
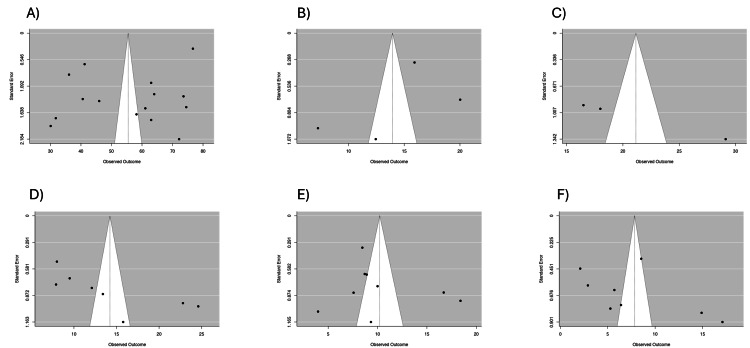
Funnel plots Oswestry disability index (ODI) A) baseline; B) up to 1 week post-operation; C) 1 month post-operation; D) 3 months post-operation; E) 6 months post-operation; F) 12 months post-operation

**Figure 8:**
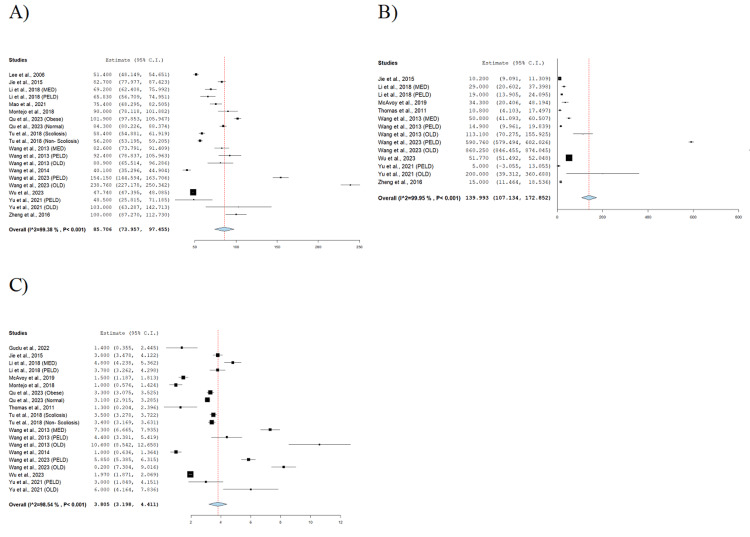
Perioperative outcomes A) perioperative time; B) estimated intraoperative blood loss; C) length of hospital stay Reference: Guclu et al., 2022 [22]; Lee et al., 2006 [24]; Jie et al., 2015 [26]; Li et al., 2018 (MED) [25]; Li et al., 2018 (PELD) [25]; Mao et al., 2021 [28]; McAvoy et al., 2019 [29]; Montejo et al., 2018 [30]; Qu et al., 2023 (Obese) [31]; Qu et al., 2023 (Normal) [31]; Thomas et al., 2011 [32]; Tu et al., 2018 (Scoliosis) [33]; Tu et al., 2018 (Non-Scoliosis) [33]; Wang et al., 2013 (MED) [34]; Wang et al., 2013 (PELD) [34]; Wang et al., 2013 (OLD) [34]; Wang et al., 2014 [35]; Wang et al., 2023 (PELD) [41]; Wang et al., 2023 (OLD) [41]; Wu et al., 2023 [36]; Yu et al., 2021 (PELD) [38]; Yu et al., 2021 (OLD) [38]; Zheng et al., 2016 [40] PELD: percutaneous endoscopic lumbar discectomy; MED: microendoscopic discectomy; OLD: open laminectomy discectomy

**Figure 9:**
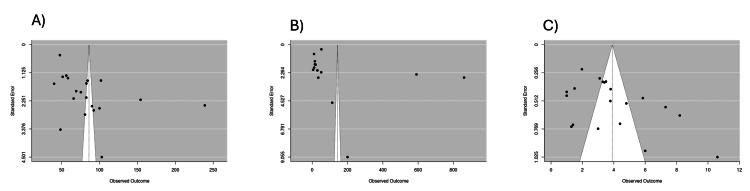
Funnel plots perioperative outcomes A) perioperative time; B) estimated intraoperative blood loss; C) length of hospital stay

**Figure 10:**
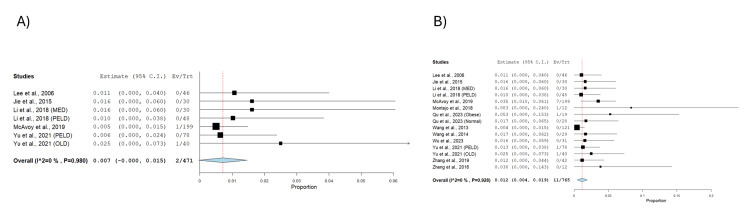
Postoperative outcomes A) lumber fusion requirement; B) reoperation of the same level Reference: Lee et al., 2006 [24]; Jie et al., 2015 [26]; Li et al., 2018 (MED) [25]; Li et al., 2018 (PELD) [25]; McAvoy et al., 2019 [29]; Montejo et al., 2018 [30]; Qu et al., 2023 (Obese) [31]; Qu et al., 2023 (Normal) [31]; Wang et al., 2013 [34]; Wang et al., 2014 [35]; Wu et al., 2023 [36]; Yu et al., 2021 (PELD) [38]; Yu et al., 2021 (OLD) [38]; Zhang et al., 2019 [39]; Zheng et al., 2016 [40] PELD: percutaneous endoscopic lumbar discectomy; MED: microendoscopic discectomy; OLD: open laminectomy discectomy

**Figure 11:**
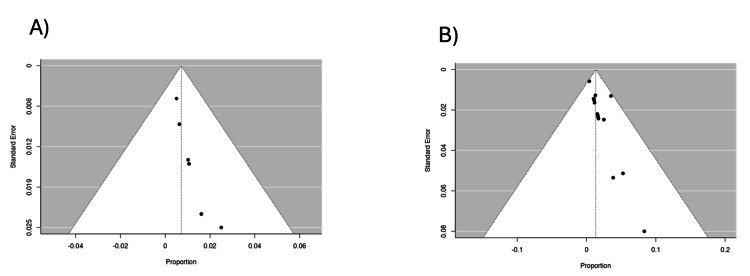
Funnel plots postoperative outcomes A) lumber fusion requirement; B) reoperation of the same level

**Figure 12:**
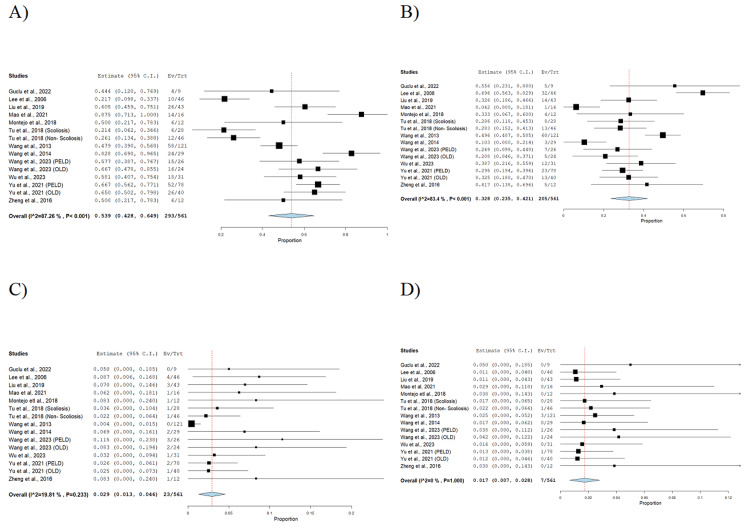
MacNab criteria A) excellent; B) good; C) fair; D) poor Reference: Guclu et al., 2022 [22]; Lee et al., 2006 [24]; Liu et al., 2019 [27]; Mao et al., 2021 [28]; Montejo et al., 2018 [30]; Tu et al., 2018 (Scoliosis) [33]; Tu et al., 2018 (Non-Scoliosis) [33]; Wang et al., 2013 [34]; Wang et al., 2014 [35]; Wang et al., 2023 (PELD) [41]; Wang et al., 2023 (OLD) [41]; Wu et al., 2023 [36]; Yu et al., 2021 (PELD) [38]; Yu et al., 2021 (OLD) [38]; Zheng et al., 2016 [40] PELD: percutaneous endoscopic lumbar discectomy; MED: microendoscopic discectomy; OLD: open laminectomy discectomy

**Figure 13:**
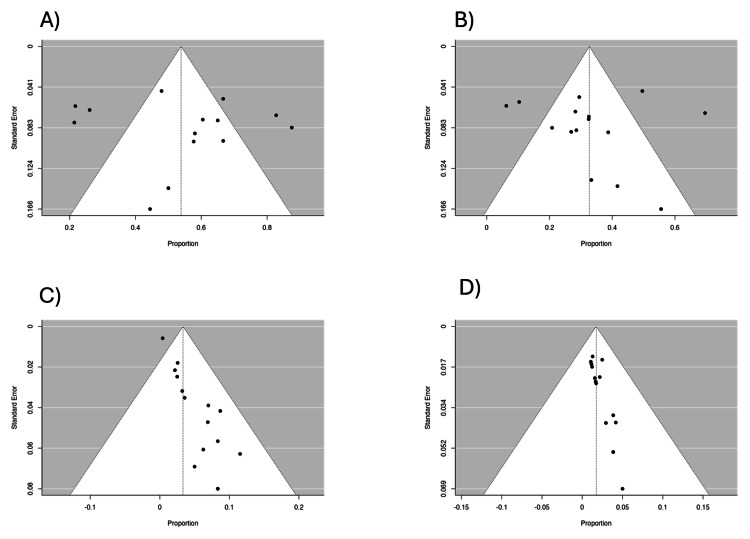
Funnel plots MacNab criteria A) excellent; B) good; C) fair; D) poor

## Appendices

Supplemental material 1: actual search strategies

PubMed

Lumbar Discectomy OR "Lumbar Discectom*" OR "Lumbar Diskectom*" OR lumbar microdiscectomy OR "lumbar microdiscectom*" OR "lumbar microdiskectom*"

 (("lumbarised"[All Fields] OR "lumbarization"[All Fields] OR "lumbarized"[All Fields] OR "lumbars"[All Fields] OR "lumbosacral region"[MeSH Terms] OR ("lumbosacral"[All Fields] AND "region"[All Fields]) OR "lumbosacral region"[All Fields] OR "lumbar"[All Fields]) AND ("diskectomy"[MeSH Terms] OR "diskectomy"[All Fields] OR "discectomies"[All Fields] OR "discectomy"[All Fields])) OR "lumbar discectom*"[All Fields] OR "lumbar diskectom*"[All Fields] OR (("lumbarised"[All Fields] OR "lumbarization"[All Fields] OR "lumbarized"[All Fields] OR "lumbars"[All Fields] OR "lumbosacral region"[MeSH Terms] OR ("lumbosacral"[All Fields] AND "region"[All Fields]) OR "lumbosacral region"[All Fields] OR "lumbar"[All Fields]) AND ("microdiscectomies"[All Fields] OR "microdiscectomy"[All Fields])) OR "lumbar microdiscectom*"[All Fields] OR "lumbar microdiskectom*"[All Fields]

6182

AND

Infan* OR newborn* OR new-born* OR perinat* OR neonat* OR baby OR baby* OR babies OR toddler* OR "minor" OR minors* OR "boy" OR "boys" OR boyfriend OR boyhood OR girl* OR kid OR kids OR child OR child* OR children* OR schoolchild* OR schoolchild OR school child [tiab] OR school child*[tiab] OR adolescen* OR juvenil* OR youth* OR teen* OR underage* OR "under age" OR "under aged" OR pubescen* OR pediatrics[mh] OR pediatric* OR paediatric* OR peadiatric* OR prematur* OR preterm*

 "infan*"[All Fields] OR "newborn*"[All Fields] OR "new born*"[All Fields] OR "perinat*"[All Fields] OR "neonat*"[All Fields] OR

("infant, newborn"[MeSH Terms] OR ("infant"[All Fields] AND "newborn"[All Fields]) OR "newborn infant"[All Fields] OR "baby"[All Fields] OR "infant"[MeSH Terms] OR "infant"[All Fields]) OR "baby*"[All Fields] OR ("baby s"[All Fields] OR "babys"[All Fields] OR "infant"[MeSH Terms] OR "infant"[All Fields] OR "babies"[All Fields]) OR "toddler*"[All Fields] OR "minor"[All Fields] OR "minors*"[All Fields] OR "boy"[All Fields] OR "boys"[All Fields] OR ("boyfriend"[All Fields] OR "boyfriend s"[All Fields] OR "boyfriends"[All Fields]) OR "boyhood"[All Fields] OR "girl*"[All Fields] OR "kid"[All Fields] OR "kids"[All Fields] OR ("child"[MeSH Terms] OR "child"[All Fields] OR "children"[All Fields] OR "child s"[All Fields] OR "children s"[All Fields] OR "childrens"[All Fields] OR "childs"[All Fields]) OR "child*"[All Fields] OR "children*"[All Fields] OR "schoolchild*"[All Fields] OR "schoolchild"[All Fields] OR "school child"[Title/Abstract] OR "school child*"[Title/Abstract] OR "adolescen*"[All Fields] OR "juvenil*"[All Fields] OR "youth*"[All Fields] OR "teen*"[All Fields] OR "underage*"[All Fields] OR "under age"[All Fields] OR "under aged"[All Fields] OR "pubescen*"[All Fields] OR "pediatrics"[MeSH Terms] OR "pediatric*"[All Fields] OR "paediatric*"[All Fields] OR "peadiatric*"[All Fields] OR "prematur*"[All Fields] OR "preterm*"[All Fields]

5869252

 ((("lumbarised"[All Fields] OR "lumbarization"[All Fields] OR "lumbarized"[All Fields] OR "lumbars"[All Fields] OR "lumbosacral region"[MeSH Terms] OR ("lumbosacral"[All Fields] AND "region"[All Fields]) OR "lumbosacral region"[All Fields] OR "lumbar"[All Fields]) AND ("diskectomy"[MeSH Terms] OR "diskectomy"[All Fields] OR "discectomies"[All Fields] OR "discectomy"[All Fields])) OR "lumbar discectom*"[All Fields] OR "lumbar diskectom*"[All Fields] OR (("lumbarised"[All Fields] OR "lumbarization"[All Fields] OR "lumbarized"[All Fields] OR "lumbars"[All Fields] OR "lumbosacral region"[MeSH Terms] OR ("lumbosacral"[All Fields] AND "region"[All Fields]) OR "lumbosacral region"[All Fields] OR "lumbar"[All Fields]) AND ("microdiscectomies"[All Fields] OR "microdiscectomy"[All Fields])) OR "lumbar microdiscectom*"[All Fields] OR "lumbar microdiskectom*"[All Fields]) AND ("infan*"[All Fields] OR "newborn*"[All Fields] OR "new born*"[All Fields] OR "perinat*"[All Fields] OR "neonat*"[All Fields] OR ("infant, newborn"[MeSH Terms] OR ("infant"[All Fields] AND "newborn"[All Fields]) OR "newborn infant"[All Fields] OR "baby"[All Fields] OR "infant"[MeSH Terms] OR "infant"[All Fields]) OR "baby*"[All Fields] OR ("baby s"[All Fields] OR "babys"[All Fields] OR "infant"[MeSH Terms] OR "infant"[All Fields] OR "babies"[All Fields]) OR "toddler*"[All Fields] OR "minor"[All Fields] OR "minors*"[All Fields] OR "boy"[All Fields] OR "boys"[All Fields] OR ("boyfriend"[All Fields] OR "boyfriend s"[All Fields] OR "boyfriends"[All Fields]) OR "boyhood"[All Fields] OR "girl*"[All Fields] OR "kid"[All Fields] OR "kids"[All Fields] OR ("child"[MeSH Terms] OR "child"[All Fields] OR "children"[All Fields] OR "child s"[All Fields] OR "children s"[All Fields] OR "childrens"[All Fields] OR "childs"[All Fields]) OR "child*"[All Fields] OR "children*"[All Fields] OR "schoolchild*"[All Fields] OR "schoolchild"[All Fields] OR "school child"[Title/Abstract] OR "school child*"[Title/Abstract] OR "adolescen*"[All Fields] OR "juvenil*"[All Fields] OR "youth*"[All Fields] OR "teen*"[All Fields] OR "underage*"[All Fields] OR "under age"[All Fields] OR "under aged"[All Fields] OR "pubescen*"[All Fields] OR "pediatrics"[MeSH Terms] OR "pediatric*"[All Fields] OR "paediatric*"[All Fields] OR "peadiatric*"[All Fields] OR "prematur*"[All Fields] OR "preterm*"[All Fields])

Result 843

Embase

Session Results

.......................................................

No.  Query Results                                          Results  Date      

#17. #9 AND #16                                                 879  15 Sep 2023

#16. #10 OR #11 OR #12 OR #13 OR #14 OR #15               6,989,112  15 Sep 2023

#15. infan* OR newborn* OR 'new born*' OR perinat* OR     6,989,112  15 Sep 2023

     neonat* OR baby OR baby* OR babies OR toddler* OR

     'minor' OR minors* OR 'boy' OR 'boys' OR

     boyfriend OR boyhood OR girl* OR kid OR kids OR

     child OR child* OR children* OR schoolchild* OR

     schoolchild OR adolescen* OR juvenil* OR youth*

     OR teen* OR underage* OR 'under age' OR 'under

     aged' OR pubescen* OR pediatrics OR pediatric* OR

     paediatric* OR peadiatric* OR prematur* OR

     preterm*

#14. 'pediatrics'/exp OR pediatrics                       1,057,942  15 Sep 2023

#13. 'baby'/exp OR baby                                      83,825  15 Sep 2023

#12. 'infant'/exp OR infant                               1,406,210  15 Sep 2023

#11. 'adolescent'/exp OR adolescent                       2,061,566  15 Sep 2023

#10. 'child'/exp OR child                                 3,890,708  15 Sep 2023

#9.  #1 OR #2 OR #3 OR #4 OR #5 OR #6 OR #7 OR #8             8,708  15 Sep 2023

#8.  'lumbar microdiskectom*'                                    34  15 Sep 2023

#7.  'lumbar microdiscectom*'                                   571  15 Sep 2023

#6.  'lumbar micro-discectomy' OR 'lumbar                       585  15 Sep 2023

     microdiskectomy' OR 'lumbar microdiscectomy'

#5.  'lumbar microdiscectomy'/exp OR 'lumbar                  1,420  15 Sep 2023

     microdiscectomy' OR (lumbar AND

     ('microdiscectomy'/exp OR microdiscectomy))

#4.  'lumbar diskectom*'                                        170  15 Sep 2023

#3.  'lumbar discectom*'                                      2,400  15 Sep 2023

#2.  'lumbar diskectomy' OR 'lumbar discectomy'               2,496  15 Sep 2023

#1.  'lumbar discectomy'/exp OR 'lumbar discectomy' OR        8,431  15 Sep 2023

     (lumbar AND ('discectomy'/exp OR discectomy))

Cochrane Library

Search Name:

Date Run:        15/09/2023 12:34:32

Comment:

ID        Search Hits

#1        (Lumbar Discectomy OR Lumbar Diskectomy OR lumbar microdiscectomy  OR lumbar microdiskectom):ti,ab,kw (Word variations have been searched)           1103

#2        MeSH descriptor: [Child] explode all trees    78477

#3        MeSH descriptor: [Adolescent] explode all trees      125806

#4        MeSH descriptor: [Infant] explode all trees   41997

#5        (Infant OR Infan* OR newborn* OR new-born* OR perinat* OR neonat* OR baby OR baby* OR babies OR toddler* OR "minor" OR minors* OR "boy" OR "boys" OR boyfriend OR boyhood OR girl* OR kid OR kids OR child OR child* OR children* OR schoolchild* OR schoolchild OR school child OR school child* OR adolescen* OR juvenil* OR youth* OR teen* OR underage* OR “under age” OR “under aged” OR pubescen* OR pediatrics OR pediatric* OR paediatric* OR peadiatric* OR prematur* OR preterm*):ti,ab,kw (Word variations have been searched)    387858

#6        {OR #2-#5}    387858

#7        #1 AND #6     102

Scopus

( TITLE-ABS-KEY ( infant OR infan* OR newborn* OR new-born* OR perinat* OR neonat* OR baby OR baby* OR babies OR toddler* OR "minor" OR minors* OR "boy" OR "boys" OR boyfriend OR boyhood OR girl* OR kid OR kids OR child OR child* OR children* OR schoolchild* OR schoolchild OR "school child" OR "school child*" OR adolescen* OR juvenil* OR youth* OR teen* OR underage* OR "under age" OR "under aged" OR pubescen* OR pediatrics OR pediatric* OR paediatric* OR peadiatric* OR prematur* OR preterm* ) ) AND ( TITLE-ABS-KEY ( "Lumbar Discectom*" OR "Lumbar Diskectom*" OR "lumbar microdiscectom*" OR "lumbar microdiskectom*" ) )

361

.......................................................

Web of Science

Web of Science Search Strategy (v0.1)

# Database: Web of Science Core Collection

# Entitlements:

- WOS.IC: 1993 to 2023

- WOS.CCR: 1985 to 2023

- WOS.SCI: 1900 to 2023

- WOS.AHCI: 1975 to 2023

- WOS.BHCI: 2005 to 2023

- WOS.BSCI: 2005 to 2023

- WOS.ESCI: 2005 to 2023

- WOS.ISTP: 1990 to 2023

- WOS.SSCI: 1900 to 2023

- WOS.ISSHP: 1990 to 2023

# Searches:

1: TS=(“Lumbar Discectom*” OR “Lumbar Diskectom*” OR “lumbar microdiscectom*”  OR “lumbar microdiskectom*”)                                               Date Run: Fri Sep 15 2023 21:36:55 GMT+1000 (Australian Eastern Standard Time)                   Results: 2496

2: TS=(Infant OR Infan* OR newborn* OR new-born* OR perinat* OR neonat* OR baby OR baby* OR babies OR toddler* OR "minor" OR minors* OR "boy" OR "boys" OR boyfriend OR boyhood OR girl* OR kid OR kids OR child OR child* OR children* OR schoolchild* OR schoolchild OR “school child” OR “school child*” OR adolescen* OR juvenil* OR youth* OR teen* OR underage* OR “under age” OR “under aged” OR pubescen* OR pediatrics OR pediatric* OR paediatric* OR peadiatric* OR prematur* OR preterm*)                                            Date Run: Fri Sep 15 2023 21:38:21 GMT+1000 (Australian Eastern Standard Time)               Results: 4596603

3: #2 AND #1                                     Date Run: Fri Sep 15 2023 21:39:04 GMT+1000 (Australian Eastern Standard Time)               Results: 102

Supplemental material 2

Supplemental material 3

Supplemental material 4

Supplemental material 5

Supplemental material 6

Supplemental material 7

Supplemental material 8

Supplemental material 9

Supplemental material 10

Supplemental material 11

Supplemental material 12

Supplemental material 13
